# Dengue Seroprevalence of Healthy Adults in Singapore: Serosurvey among Blood Donors, 2009

**DOI:** 10.4269/ajtmh.14-0671

**Published:** 2015-07-08

**Authors:** Swee-Ling Low, Sally Lam, Wing-Yan Wong, Diana Teo, Lee-Ching Ng, Li-Kiang Tan

**Affiliations:** Environmental Health Institute, National Environment Agency, Singapore; Blood Services Group, Health Sciences Authority, Singapore

## Abstract

Routine national notifications of dengue cases typically do not reflect the true dengue situation due to large proportion of unreported cases. Serosurveys, when conducted periodically, could shed light on the true dengue infections in the population. To determine the magnitude of dengue infections of the adult population in Singapore following the outbreak in 2007, we performed a cross-sectional study on blood donor samples from December 2009 to February 2010. The residual blood of 3,995 donors (aged 16–60 years) was screened for the presence of dengue-specific immunoglobulin G (IgG) and IgM using enzyme-linked immunosorbent assay (ELISA) kits. The age-weighted IgG prevalence of residents was 50.8% (*N* = 3,627, 95% confidence interval [CI] = 49.4–52.3%). Dengue IgG prevalence increased with age, with the lowest in 16–20 years age group (16.1%) and the highest in 56–60 years age group (86.6%). Plaque reduction neutralization test (PRNT) on samples of young resident adults (aged 16–30 years) revealed lower prevalence of neutralizing antibodies to each serotype, ranging from 5.4% to 20.3% compared with the older age groups. The level of exposure to dengue among the young adults is relatively low despite the endemicity of the disease in Singapore. It partially explains the population’s susceptibility to explosive outbreaks and the high incidence rate among young adults.

## Introduction

Dengue is an important mosquito-borne viral infection caused by four closely related serotypes of dengue virus (DENV-1, DENV-2, DENV-3, and DENV-4). Infection by DENV can be asymptomatic or can inflict a vast spectrum of clinical illness ranging from undifferentiated fever, dengue fever (DF), dengue hemorrhagic fever (DHF) to dengue shock syndrome (DSS). The World Health Organization (WHO) estimates that almost half of the global population is living in countries where dengue is endemic. During the past 50 years, global dengue incidences have increased by 30-fold. This fast-growing infectious disease is projected to cause some 50–100 million new infections annually in more than 100 endemic countries, leading to half a million hospitalizations.^1^–^3^

The iceberg analogy of dengue notification is well known. Passive surveillance through case notifications does not reflect the true dengue situation in a population. Cohort studies in different provinces of Thailand and in Nicaragua had revealed higher numbers of prospectively determined dengue incidences as compared with national reported figures, with a discrepancy of 8- to 21.3-folds.^4^,^5^ High proportion (up to 87%) of inapparent DENV infection in children^6^ and older adults^7^ has been reported in Thailand and Singapore, respectively. Seroprevalence studies could provide a more accurate estimate of DENV infections in a population.

Dengue is endemic in Singapore. The first incidence of DHF was reported in 1966 and DF became a notifiable disease in 1977. A nationwide *Aedes* mosquito control program that incorporated source reduction, public health education, and law enforcement was introduced in the 1970s. This active control program led to a period of low dengue incidence over the next decade.^8^ In the late 1980s, Singapore experienced a resurgence of dengue cases despite the low *Aedes* premise index of around 1.^9^ Incidence rate rose from 16.7 cases per 100,000 population in 1987 to 322.5 in the 2005 epidemic. The incidence of dengue appears to follow a 6-year cycle of increasing incidence with peaks in 1992, 1998, and 2004–2005.^10^,^11^ Following a lull period in 2006 (dengue incidence of 63.4 cases per 100,000 population),^12^ Singapore experienced another epidemic in 2007 when the dengue incidence rate hit 180.6 cases per 100,000 population.^13^ Although all four DENV serotypes are co-circulating in Singapore, DENV-1 and DENV-2 were the predominant serotypes in outbreaks of 2004–2005 and 2007, respectively.

Surveillance and seroepidemiological data have revealed that, starting from the late 1980s, dengue has evolved from a childhood illness to a disease of adult predominance. Along with the low incidence from 1970s to 1980s, the median age of DENV infection has progressively shifted from 14 years in 1973 to 28 years in 1994 and 31 years in 2005 to 37 years in 2007.^9^,^14^ The age shift is most likely the result of declining herd immunity among adults, evident from the progressive decline in dengue prevalence among youth and adults (aged 15–39 years) from 70–86% in 1982–1984^8^ to 13.1–62.6% in 1998.^15^

Limited information is available on the profiles of dengue serotype-specific neutralizing antibody, which is essential for in-depth understanding of the dengue herd immunity in Singapore population. Only two local serosurveys had reported the level of serotype reactivity in dengue immunoglobulin G (IgG)–seropositive samples by plaque reduction neutralization test (PRNT).^16^,^17^ Chow and others^17^ demonstrated that about 60% (23 out of 38 dengue IgG seropositives) of young adults aged 19–26 years had neutralizing antibodies against DENV-2 in 1998–2000 whereas Wilder-Smith and others^16^ revealed all four DENV serotypes circulated in Singapore and DENV-3 was the most prevalent serotype among 164 healthy adult volunteers (aged 18–30 years) in 2004.

To provide a holistic view on the level of exposure to dengue in the adult population of Singapore following the 2007 outbreak, we conducted a cross-sectional study using the residual blood samples collected from healthy blood donors from December 2009 to February 2010.

## Materials and Methods

### Sample selection.

In this study, residual blood samples were collected from blood donors by the Blood Services Group, Health Sciences Authority (HSA) from December 2009 to February 2010. A total of 12,000 residual blood samples were collected during this period. An initial sample size of 4,000 for the study was determined, based on estimated seroprevalence of 59%^18^ at a confidence level of 99% and precision of 2%. Sample selection was based on stratified random sampling technique according to demography of adult population in year 2009 (Department of Statistics, Income, Expenditure and Population Statistics Division, Singapore). Samples were randomly selected using the Statistical Package for Social Sciences (SPSS) software version 19 (SPSS Inc., Chicago, IL) with a sample size of 350–500 in per age group. A final sample number of 3,995 was used in this study. Serum from blood samples was transferred to sterile 2-mL cryo-tubes and stored at −20°C until testing.

### Ethics statement.

This study was reviewed and approved by the HSA management committee and the National Environment Agency’s Environmental Health Institute management committee with bioethics considerations. Consent to test the samples for infectious diseases affecting the blood supply was obtained from the blood donors as part of the Donor Health Assessment Questionnaire Form. All donor identifiers were removed prior to the testing of samples, and only the age, gender, residency status, and first three-digit residential postal codes were used in the analysis of results.

### Serology tests.

Serum samples were tested for dengue-specific IgG and IgM by Panbio Dengue IgG Indirect enzyme-linked immunosorbent assay (ELISA) and IgM Capture ELISA (Alere Inc., Waltham, MA), respectively. The kits were used according to the manufacturer’s instruction. A positive ELISA result was defined as having > 11 Panbio units. To confirm that the IgM antibody detected was truly dengue specific, samples testing positive in the Panbio IgM Capture ELISA were also tested with the commercial IgM Capture ELISA from Standard Diagnostics (SD) Inc. (Yongin-si, Gyeonggi-do, Korea) according to the manufacturer’s instructions. Samples that were positive for only IgM antibody by the Panbio ELISA were tested with PRNT for the presence of DENV-neutralizing antibodies.

### Plaque reduction neutralization test.

Thirty IgG-seropositive samples from each age group of 16–20, 21–25, 26–30, 31–35, 36–40, and 56–60 years were randomly selected and tested for the presence of neutralizing antibodies to DENV serotypes using PRNT. For each DENV serotype, two local strains of different genotypes were used (Table 1).

The PRNT was adapted from Morens and others.^19^ Briefly, heat-inactivated sera were serially diluted into 1:10, 1:100, and 1:1,000 using minimum essential medium (GE Healthcare, Little Chalfont, Buckinghamshire, United Kingdom) supplemented with 3% fetal bovine serum, l-glutamine, sodium pyruvate, and penicillin–streptomycin. One hundred microliters of each diluted sera and DENV (containing around 800 pfu/mL) were added into the wells of 96-well plate. This step was repeated for each DENV strain. The virus–antibody binding reaction was allowed to take place during an hour incubation at 37°C. Baby hamster kidney (BHK) cells were seeded at 1 × 10^5^ cells/mL in 24-well plates for at least an hour before use. Fifty microliters of the virus–antibody mixture was added into the wells containing BHK cells in triplicates. The plates were then incubated overnight at 37°C to allow virus adsorption onto BHK cells and overlaid with the complete carboxymethyl cellulose (CMC) medium. After 4 days of incubation at 37°C, visualization of the plaques was done by fixing the cells with 20% formalin and staining them using the naphthol blue stain solution. Plaques were counted and calculations of 50% end point plaque reduction neutralization titers were computed using log probit paper by the method of Russell and others.^20^ Antibody titers were expressed as the reciprocal of the end point dilution. Samples with PRNT_50_ titers ≥ 30 were considered to have neutralizing antibody against DENV.

### Data analysis.

Among the 3,995 randomly selected samples, only the results of samples obtained from residents (Singaporeans and Permanent Residents of Singapore, *N* = 3,627) were analyzed. The dengue IgG prevalence among residents was weighted to adjust for age and gender distribution in the population. The sampling weights were prepared using the 2009 demographic data of resident population as mentioned above. Data analysis was performed using SPSS version 19 (SPSS Inc.) and Microsoft Excel 2007 (Microsoft Corporation, Redmond, WA). χ^2^ test or *z*-test, where appropriate, was used to compare the significance of differences in seropositivity between groups. Odds ratios (OR) and their 95% confidence interval (95% CI) were estimated to indicate the probability of past DENV infection for each group as compared with a referent. Probability (*P*) values < 0.05 were considered statistically significant.

The prevalence of antibodies reactive to single, multiple DENV serotype/s, and each serotype was obtained by multiplying the proportion of IgG-positive subjects with neutralizing antibodies to one or more DENV serotypes and the weighted dengue IgG prevalence of each respective age group.

## Results

### Demographic data.

Among residents, 1,874 (51.7%) were female and 1,753 (48.3%) were male. Of these, 355 (9.8%) were in the age group of 16–20 years, 304 (8.4%) were 21–25 years, 357 (9.8%) were 26–30 years, 402 (11.1%) were 31–35 years, 446 (12.3%) were 36–40 years, 482 (13.3%) were 41–45 years, 484 (13.3%) were 46–50 years, 446 (12.3%) were 51–55 years, and 351 (9.7%) were 56–60 years.

### Dengue-specific IgG results.

Among residents, 1,885 (52%, 95% CI = 50.3–53.6%) had dengue IgG, indicating past DENV infection (Table 2). The age weighted dengue IgG prevalence was 50.8% (95% CI = 49.4–52.3%). The increase of dengue IgG prevalence with age is in agreement with previous studies using similar detection assays.^7^,^18^ Residents in the 16–20 and 21–25 age groups (16.1% each) presented the lowest IgG prevalence, followed by 33.3% in the 26–30 age group, 43.2% in the 31–35 age group, 48.4% in the 36–40 age group, 57.3% in the 41–45 age group, 69.6% in the 46–50 age group, 79.6% in the 51–55 age group, and 86.6% in the 56–60 age group. In residents above 20 years of age, the OR of dengue IgG prevalence showed increment of 1.03–2.48 for every 5 years increase in age. Dengue IgG prevalence was significantly higher in males (52.7%) than females (49.0%) (*P* = 0.011). In the OR analysis, males were 1.21 times more likely to have exposed to dengue than females.

### Dengue-specific IgM results.

Panbio Dengue IgM Capture ELISA tests revealed that 2.83% (113 out of 3,995) of samples were positive. Of these, 35 samples were tested positive for only dengue-specific IgM, and 78 were tested positive for both IgM and IgG (probable recent DENV infection). All of the 35 samples testing positive for only dengue-specific IgM in the Panbio Dengue IgM Capture ELISA were tested negative in the SD Dengue IgM Capture ELISA. They also contained no neutralizing antibodies to all four DENV serotypes. Among the 78 samples testing positive for both IgM and IgG in the Panbio ELISAs, only 10 showed the presence of dengue-specific IgM in SD Dengue IgM Capture ELISA. Because of the presence of IgG in these samples, PRNT was not done to validate the presence of IgM. These results suggest that almost all of the 113 IgM-positive samples were likely to be false-positive results.

### DENV-neutralizing antibody profiles.

**Figure 1.**
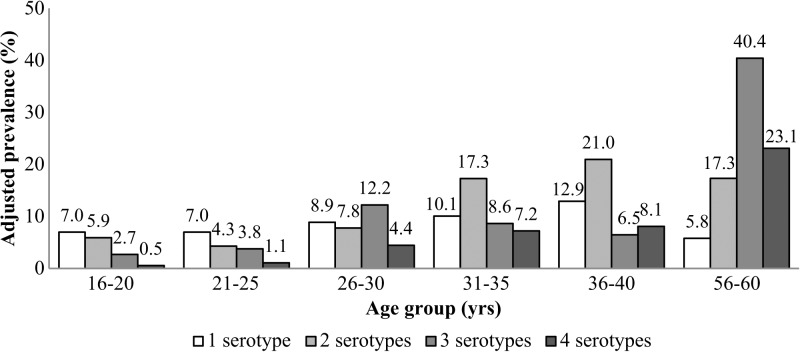
Prevalence of antibodies reactive to single and multiple dengue virus (DENV) serotypes in different age groups (*N* = 30 per age group). The prevalence for single and multiple serotypes in each age group was obtained by multiplying the proportion of subjects with antibodies reactive to one or more DENV serotypes and the weighted dengue prevalence of each respective age group.

DENV-neutralizing antibody profiles showed monotypic PRNT pattern in most young adult residents (suggesting single infection), and an increasing proportion showing multitypic PRNT pattern with increasing age (Figure 1

). The majority of young adults (aged 16–25 years) had antibodies reactive to a single dengue serotype (7.0% of cohort population), followed by those with dual serotypes (4.3–5.9%). In general, adults who were 26 years of age and above showed high prevalence to multiple serotypes, with those aged 56–60 years having the highest prevalence to three or more serotypes (23.1–40.4%).

**Figure 2.**
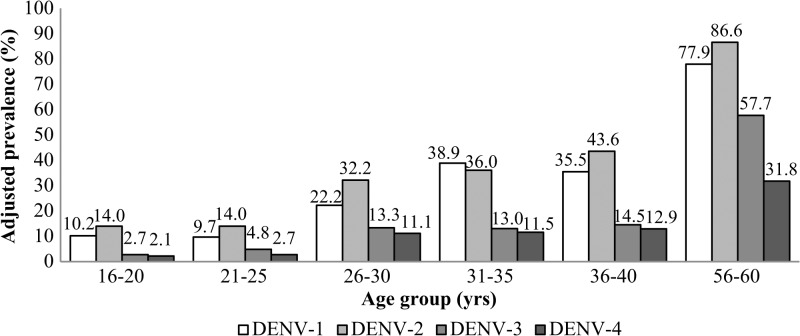
Prevalence of antibodies reactive to each dengue virus (DENV) serotype in different age groups (*N* = 30 per age group). The prevalence to each DENV serotype was obtained by multiplying the proportion of immunoglobulin G (IgG)–positive subjects with DENV-neutralizing antibodies and the weighted dengue prevalence of each respective age group.

The prevalence of antibodies reactive to each DENV serotype generally increases with age (Figure 2

), with an adjusted prevalence range of 9.7–77.9% for DENV-1, 14.0–86.6% for DENV-2, 2.7–57.7% for DENV-3, and 2.1–31.8% for DENV-4. Compared with older age groups, young residents of age 16–30 years showed lower prevalence of neutralizing antibodies to each serotype, 14.2%, 20.3%, 7.1%, and 5.4% to DENV-1, DENV-2, DENV-3, and DENV-4, respectively. PRNT_50_ titers for selected dengue IgG-positive samples are presented in Supplemental Table 1.

## Discussion

Seroprevalence studies are essential for understanding the true burden of disease in the population. When conducted regularly, the information generated contributes to the evaluation of the efficacy of control programs. Seroprevalence data are also currently used in mathematical models that aim to estimate the impact of potential vaccine implementation.

This study analyzed residual samples collected from healthy blood donors between 16 and 60 years of age, which represents a subset of the population and may therefore not be representative of the entire adult population in Singapore. However, we used a relatively large sample size in the study and observed a similar increasing trend of dengue IgG prevalence with age, which is consistent with previous serosurveys conducted in Singapore.^7^,^18^ To ensure that the geographic distribution of subjects was representative of the population, the (first three) postal codes of the subjects’ residential addresses were mapped against the district and geographical location using Geographical Information System (GIS) software (ESRI ArcGIS 10.1, Redlands, CA) and shown to be widely distributed in populated areas (data not shown).

Our study indicates that 50.8% of the Singapore residents aged 16–60 years had past DENV infection, which shows a lower prevalence rate compared with the National Health Survey (NHS) conducted in 2004 (59%).^21^ A contributing factor to this decline could be the higher age range of participants involved in the NHS (aged 18–74 years), in contrast to our serosurvey on blood donors where the age range was 16–60 years. It is observed in this serosurvey that the majority of young adult in Singapore have not been exposed to dengue. As the trend of dengue IgG prevalence increases with age, it is expected to see a lower prevalence rate in this serosurvey (that involved participants of younger age) compared with the NHS.

**Figure 3.**
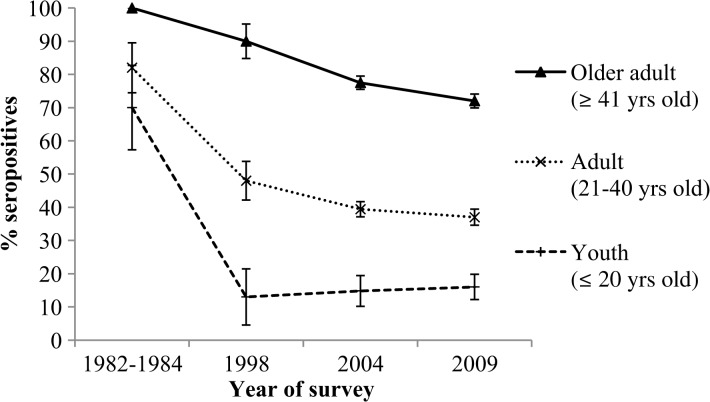
Seroprevalence data (and 95% confidence interval [CI]) of the dengue serosurveys conducted in Singapore from 1982 to 2009. For 2004 serosurvey, Youth refers to age 18–20 years; for 1982–1984 and 1998 serosurveys, Youth refers to age 15–19 years, Adult refers to 20–39 years, and Older adult refers to above 40 years. For 1982–1984 and 1998 serosurveys, hemagglutination inhibition (HI) test and dengue virus (DENV-2) antigen were used; for 2004 and 2009 serosurveys, Panbio Dengue Indirect immunoglobulin G (IgG) enzyme-linked immunosorbent assays (ELISAs) were used.

Evidence of low level of exposure to dengue in young adults of age 16–25 years was supported by their IgG prevalence of 16.1%. This trend among the young community has also been observed in the NHS, where a prevalence rate of 17.2% was reported in residents of age 18–24 years.^18^ Of greater interest is the decreasing trend of dengue seropositivity in adults over time, as seen in the serosurveys conducted in Singapore (Figure 3

). Though hemagglutination inhibition (HI) tests (DENV-2 antigen) were used in 1982–1984 and 1998 serosurveys, the Panbio Indirect IgG ELISA has been used in all subsequent studies, including the current one. The sensitivity, specificity, and agreement rate between HI and ELISA has previously been reported to be good.^22^,^23^ Chungel and others^22^ reported the coefficients of correlation between IgG ELISA and HI results to be highly significant. Likewise, McBride and others demonstrated that the sensitivity and specificity of the ELISA was 99.17% and 96.18%, respectively, when compared with HI test.^23^ It is thus reasonable to make inferences on the trends of dengue seroprevalence in Singapore based on these studies.

In the 1982–1984 serosurvey, HI test against DENV-2 revealed that 70% of youths (aged ≤ 20 years) have previously been infected with dengue.^8^ A sharp decrease in seropositivity was observed in youths in 1998, using a similar assay. Thereafter, the prevalence level has remained below 20% in the 2000s (NHS and this study).^15^,^18^ Similarly, the percentage of seropositivity in the age group 21–40 years was also substantially reduced by about two times, from 82% in the 1980s to about 40% in the 2000s.^8^,^18^ The declining seroprevalence likely reflects the reduction in disease burden—a consequence of the intensive vector control over decades. This has led to a population with low herd immunity, which explains its vulnerability to outbreaks despite a low *Aedes* premise index of < 1%.

The decreasing seroprevalence among adults also partially explains the age shift of dengue incidence from a median of 14 years in 1973 to above 30 years in the last decade. The incidence rates in Singapore are highest among adults.^11^,^13^,^24^,^25^ However, the gradual increase in seroprevalence with age, as revealed by this and a previous study that included children,^26^ suggests similar risk of infection among various age groups. The apparent low incidence in children could be due to milder clinical manifestations in primary infections among children as compared with adults.^27^

It is intriguing that our serosurvey presents no obvious surge in seroprevalence rate compared with the NHS study, despite two large outbreaks of more than 14,000 cases in 2005 and more than 7,000 cases in 2007. Instead, there was a decrease in prevalence within each age group, and which is most obvious among young adults. This suggests that the large outbreaks of dengue did not increase the seroprevalence of each cohort to the same levels seen in their predecessor in the same age group. This is corroborated by another study conducted in early 2014 (our unpublished data) following the 2013 outbreak of more than 22,000 cases, where the seroprevalence among those aged 16–25 years remained low at 16%.

In general, the dengue IgG prevalence of young adults in Singapore (aged 16–25 years) is much lower (16.1%) in comparison to those of younger or similar age in other endemic countries. The level of exposure to dengue was 34% in those aged 7–17 years in Sri Lanka,^28^ 65.7% in children aged 7–14 years in southern Vietnam,^29^ 79% in those aged 15–24 years in Mexico,^30^ 91% in Nicaraguan children aged 4–16 years,^31^ 97% in those < 20 years old in the Dominican Republic,^32^ and 74–91% in Brazilian children aged 5–14 years.^33^ Compared with a seropositivity of 80.3% among 35- to 44-year-old participants in a 2008 Malaysian nationwide cohort (The Malaysian Cohort),^34^ adults of similar age range in Singapore also presented with a lower level of dengue exposure (53.0%).

DENV-neutralizing antibody profiles of young residents aged 16–25 years demonstrated low levels of prevalence to all four DENV serotypes (2.7–14.1%), with the majority had antibodies reactive to only a single serotype. Using data of tested age groups, we interpolated the prevalence of DENV-1 to DENV-4 for residents (aged 16–60 years) to be 41.9%, 47.8%, 24.7%, and 16.0%, respectively. Our results show neutralizing antibody reactivity to four DENV serotypes, corroborating the virus serotype surveillance where all four DENV serotypes co-circulate in Singapore.^35^ Majority of the tested IgG-positive subjects had reactive antibodies against DENV-1 and DENV-2. This observation was anticipated because DENV-1 and DENV-2 have been the predominant serotypes in the past two decades.^36^ DENV-1 was the predominant circulating serotype in 1995–1997, 1999, and 2004–2006, whereas DENV-2 predominated in 1990–1991, 1993, 1998, 2000–2003, and 2007 onward.^10^,^16^,^35^,^36^

Intriguingly, about 4% (*N* = 7) of the 187 IgG-positive samples tested with PRNT did not show presence of neutralizing antibodies to any DENV serotypes. The Panbio dengue Indirect IgG ELISA kit might detect cross-reactive antibodies to yellow fever (YF) and Japanese encephalitis (JE).^37^ Hardly any YF incidence has been detected in this region. Furthermore, individuals vaccinated with YF (*N* = 32) showed no detectable dengue seroconversion, indicating very low cross-reactivity of the Panbio kit with YF antibodies.^38^ The product literature states that 90% of JE patients could give equivocal or positive results on dengue Indirect IgG ELISA. Although the number of JE incidence is very small in Singapore presently and there has been no national JE vaccination program, JE used to be endemic in Singapore and continues to be active in our region.^39^,^40^ Therefore, it is possible that a small percentage of positive dengue IgG results could be due to cross-reactive antibodies of other flaviviruses such as JE.

Our initial analysis showed that 2.83% (113 out of 3,995) of the study population was positive for dengue-specific IgM, but further investigations using another commercial kit and PRNT revealed that almost all of them were likely to be false-positive results. IgM can be detected in recently infected individuals and the levels can sustain till 60–90 days postinfection (convalescent phase where IgG appears). The low level of possible IgM-positive samples is most likely due to the stringent donor selection process for blood donation in Singapore, which includes a three week deferral for fever and a six month deferral after DENV infection.^41^ The outcome of the further investigation of IgM-positive samples has revealed the inappropriateness of using IgM test on healthy individuals.

## Conclusion

The data show that the level of exposure to dengue among the young adults is relatively low despite the endemicity of the disease in Singapore. It partially explains the population’s susceptibility to explosive outbreaks and the high incidence rate among young adults when compared with other age groups.

## Supplementary Material

Supplemental Table.

## Figures and Tables

**Table 1:** DENV used in PRNT

DENV serotype	Strain	Genotype
DENV-1	EHI0650Y08 (SG(EHI)DED65008, Genbank accession number: GQ357692)	Genotype I
DENV-1	EDEN203/05 (D1/SG/05K3300DK1/2005, Genbank accession number: EU081237)	Genotype III
DENV-2	EHI1170Y08 (SG(EHI)DED1171008, Genbank accession number: GQ357789)	Asian I
DENV-2	EHI0866Y07 (SG(EHI)D2/0866Y07, Genbank accession number: KP685236)	Cosmopolitan Clade II
DENV-3	EHI0040Y09 (SG(EHI)D3/0040Y09, Genbank accession number: GU370052)	Genotype I
DENV-3	EDEN219/05 (D3/SG/05K3316DK1/2005, Genbank accession number: EU081202)	Genotype III
DENV-4	EHI462Y04 (SG(EHI)D4/0462Y04, Genbank accession number: KP792536)	Genotype I
DENV-4	TCR310A129 (EHI310A129SY10, Genbank accession number: JX024758)	Genotype II

DENV = dengue virus; PRNT = plaque reduction neutralization test.

**Table 2:** Characteristics of residents (*N* = 3,627) with past DENV infection

Characteristics	No. positives/No. tested	Percentage	Weighted prevalence (%)	OR	95% CI	*P* value
Overall	1,885/3,627	52.0	50.8	–	–	–
Gender						
Female	930/1,874	49.6	49.0	1	Referent	NA
Male	955/1,753	54.5	52.7	1.21	1.07, 1.38	0.003*
Age group†						
16–20	57/355	16.1	16.1	1	NA	NA
21–25	50/304	16.4	16.1	1.03	0.68, 1.56	0.892
26–30	117/357	32.8	33.3	2.48	1.70, 3.60	< 0.05*
31–35	173/402	43.0	43.2	1.55	1.15, 2.08	0.004*
36–40	216/446	48.4	48.4	1.24	0.95, 1.63	0.115
41–45	276/482	57.3	57.3	1.43	1.10, 1.85	0.007*
46–50	337/484	69.6	69.6	1.71	1.31, 2.23	< 0.05*
51–55	355/446	79.6	79.6	1.70	1.26, 2.30	0.001*
56–60	304/351	86.6	86.6	1.66	1.13, 2.43	0.009*

CI = confidence interval; DENV = dengue virus; NA = not applicable; OR = odds ratio.

* Significant if *P* value is < 0.05.

† The preceding age group was used as the referent for calculation of the OR.
