# Supplementary material for: Dengue Seroprevalence of Healthy Adults in Singapore: Serosurvey among Blood Donors, 2009
Source: Am J Trop Med Hyg. 2015 Jul 8;93(1):40–5. doi: 10.4269/ajtmh.14-0671 (PMC4497902; doi:10.4269/ajtmh.14-0671)
Supplement: Supplementary file 1 [file SD1.pdf]

Supplemental Table 1

PRNT$_{50}$ titers of selected IgG-seropositive samples

		PRNT$_{50}$ titer								
		DENV-1		DENV-2		DENV-3		DENV-4		
Serial number	Age group	EHI0650Y08	EDEN203/05	EHI1170Y08	EHI0866Y07	EDEN219/05	EHI0040Y09	TCR310A129	EHI462Y04	Results
1	16–20	21	< 10	270	> 1,000	< 10	< 10	< 10	62	2, 4
2		< 10	< 10	< 10	< 10	< 10	< 10	< 10	< 10	Negative
3		> 1,000	800	88	16.5	< 10	< 10	< 10	< 10	1, 2
4		< 10	< 10	82	92	< 10	< 10	< 10	< 10	2
5		32	< 10	500	560	< 10	< 10	< 10	< 10	1, 2
6		> 1,000	540	160	26	48	23	< 10	13	1, 2, 3
7		15	< 10	54	58	< 10	< 10	< 10	< 10	2
8		< 10	< 10	46	300	< 10	< 10	< 10	< 10	2
9		92	< 10	29	< 10	200	740	< 10	27	1, 3
10		14.5	< 10	80	240	< 10	< 10	< 10	< 10	2
11		380	47	34	< 10	< 10	14.5	< 10	10	1, 2
12		680	130	66	66	< 10	15	< 10	11	1, 2
13		300	160	< 10	< 10	< 10	< 10	< 10	< 10	1
14		< 10	< 10	< 10	< 10	< 10	< 10	< 10	< 10	Negative
15		> 1,000	380	80	37	< 10	25	< 10	15	1, 2
16		< 10	< 10	< 10	< 10	< 10	< 10	< 10	< 10	Negative
17		34	< 10	68	17	25	90	< 10	< 10	1, 2, 3
18		19	< 10	210	470	< 10	< 10	< 10	< 10	2
19		560	420	70	18	10	18	< 10	< 10	1, 2
20		15	< 10	90	26	< 10	< 10	< 10	< 10	2
21		< 10	< 10	310	> 1,000	< 10	< 10	< 10	< 10	2
22		15	< 10	64	600	< 10	< 10	< 10	< 10	2
23		350	< 10	> 1,000	760	< 10	18	< 10	36	1, 2, 4
24		14.5	< 10	170	210	< 10	< 10	< 10	< 10	2
25		370	56	760	400	< 10	21	< 10	26	1, 2
26		84	< 10	84	15.5	< 10	19	31	88	1, 2, 4
27		> 1,000	> 1,000	96	920	120	600	18	76	1, 2, 3, 4
28		> 1,000	260	60	< 10	< 10	20	< 10	< 10	1, 2
29		520	360	28	< 10	< 10	15	< 10	< 10	1
30		< 10	< 10	760	440	< 10	< 10	< 10	< 10	2
31		520	400	19	< 10	< 10	< 10	< 10	< 10	1
32		280	115	< 10	31	< 10	16	< 10	< 10	1, 2
33		460	720	43	32	84	> 1,000	< 10	< 10	1, 2, 3
34	21–25	18	< 10	180	230	< 10	< 10	< 10	< 10	2
35		< 10	< 10	320	720	< 10	< 10	< 10	23	2
36		920	18	> 1,000	> 1,000	480	> 1,000	< 10	< 10	1, 2, 3
37		12	< 10	820	> 1,000	< 10	16	< 10	< 10	2
38		< 10	< 10	130	200	< 10	< 10	< 10	< 10	2
39		270	< 10	1,000	> 1,000	< 10	< 10	< 10	< 10	1, 2
40		19.5	< 10	390	660	< 10	< 10	< 10	< 10	2
41		< 10	< 10	82	38	< 10	< 10	< 10	< 10	2
42		> 1,000	540	52	13	< 10	22	< 10	< 10	1, 2
43		> 1,000	560	110	56	10.5	17	< 10	16	1, 2
44		270	58	580	> 1,000	< 10	34	< 10	< 10	1, 2, 3
45		350	290	880	> 1,000	19	68	38	50	1, 2, 3, 4
46		< 10	< 10	< 10	< 10	< 10	< 10	< 10	21	Negative
47		> 1,000	400	110	35	< 10	15	< 10	45	1, 2, 4
48		< 10	< 10	< 10	< 10	< 10	< 10	< 10	< 10	Negative
49		< 10	< 10	< 10	< 10	54	135	< 10	< 10	3
50		> 1,000	280	150	140	< 10	70	< 10	< 10	1, 2, 3
51		17	< 10	110	220	< 10	< 10	< 10	< 10	2
52		88	< 10	19	< 10	90	660	< 10	< 10	1, 3
53		> 1,000	310	76	72	16	35	15	21	1, 2, 3
54		45	< 10	130	250	< 10	< 10	< 10	< 10	1, 2
55		90	15	310	840	18	18	25	54	1, 2, 4
56		60	< 10	70	14	64	160	< 10	26	1, 2, 3
57		14	< 10	250	500	< 10	< 10	< 10	< 10	2
58		18	< 10	120	160	< 10	< 10	< 10	< 10	2
59		130	21	640	> 1,000	17	18	< 10	11	1, 2
60		22	< 10	13	< 10	19	13	< 10	< 10	Negative
61		540	260	16	< 10	< 10	< 10	< 10	< 10	1
62		13.5	< 10	290	620	< 10	< 10	< 10	< 10	2
63		> 1,000	720	28	< 10	< 10	< 10	< 10	< 10	1
64		35	< 10	270	370	< 10	< 10	< 10	< 10	1, 2
65		980	240	250	64	28	56	48	56	1, 2, 3, 4
66		28	< 10	74	18	< 10	< 10	24	86	2, 4
67	26–30	< 10	< 10	130	680	< 10	< 10	< 10	< 10	2

(continued)

Supplemental Table 1
Continued

		PRNT$_{50}$ titer								
		DENV-1		DENV-2		DENV-3		DENV-4		
Serial number	Age group	EHI0650Y08	EDEN203/05	EHI1170Y08	EHI0866Y07	EDEN219/05	EHI0040Y09	TCR310A129	EHI462Y04	Results
68		190	150	70	100	14	14	22	40	1, 2, 4
69		350	170	> 1,000	> 1,000	22	50	37	15	1, 2, 3, 4
70		< 10	< 10	52	115	< 10	< 10	< 10	< 10	2
71		27	16	99	310	10	16	12	< 10	2
72		920	15	> 1,000	250	> 1,000	> 1,000	28	19	1, 2, 3
73		150	46	130	200	210	390	< 10	< 10	1, 2, 3
74		19	< 10	160	110	< 10	< 10	< 10	< 10	2
75		620	88	50	12	< 10	< 10	< 10	< 10	1, 2
76		56	< 10	74	19	< 10	< 10	120	52	1, 2, 4
77		41	< 10	450	600	< 10	< 10	< 10	< 10	1, 2
78		200	66	720	> 1,000	42	190	23	12.5	1, 2, 3
79		280	78	150	140	20	100	< 10	< 10	1, 2, 3
80		19	< 10	13.5	< 10	< 10	11	19	150	4
81		29	< 10	36	13	16	360	< 10	< 10	2, 3
82		17	< 10	140	380	< 10	15	< 10	< 10	2
83		28	< 10	> 1,000	> 1,000	< 10	27	< 10	16	2
84		260	460	250	140	490	740	52	82	1, 2, 3, 4
85		120	48	140	82	< 10	46	18	64	1, 2, 3, 4
86		50	40	400	> 1,000	< 10	13	< 10	< 10	1, 2
87		> 1,000	540	64	68	16	22	< 10	< 10	1, 2
88		380	35	600	340	66	> 1,000	130	74	1, 2, 3, 4
89		< 10	< 10	16	12	< 10	< 10	21	25	Negative
90		190	18	34	< 10	< 10	< 10	< 10	12.5	1, 2
91		13	< 10	290	230	< 10	< 10	< 10	16	2
92		320	130	90	21	10	26	11.5	34	1, 2, 4
93		480	120	150	18	14.5	37	< 10	< 10	1, 2, 3
94		36	< 10	600	> 1,000	< 10	16	< 10	60	1, 2, 4
95		> 1,000	140	52	80	< 10	20.5	< 10	< 10	1, 2
96		27	10	35	72	48	47	62	24	2, 3, 4
97		115	15	> 1,000	> 1,000	< 10	46	< 10	18	1, 2, 3
98	31–35	> 1,000	22	> 1,000	> 1,000	< 10	29	32	54	1, 2, 4
99		> 1,000	320	540	> 1,000	82	60	130	56	1, 2, 3, 4
100		200	54	< 10	< 10	< 10	< 10	< 10	< 10	1
101		860	140	380	270	< 10	19	< 10	21	1, 2
102		47	< 10	130	32	< 10	15	< 10	26	1, 2
103		310	50	15	13	< 10	11	< 10	< 10	1
104		17	< 10	39	140	< 10	< 10	< 10	< 10	2
105		> 1,000	660	120	58	< 10	< 10	< 10	< 10	1, 2
106		470	680	130	160	< 10	10.5	< 10	< 10	1, 2
107		86	< 10	> 1,000	> 1,000	< 10	< 10	< 10	15	1, 2
108		47	37	560	> 1,000	< 10	< 10	< 10	11	1, 2
109		> 1,000	330	580	330	180	230	60	26	1, 2, 3, 4
110		600	760	28	15.5	< 10	< 10	< 10	12	1
111		47	19	440	> 1,000	< 10	21	< 10	12	1, 2
112		18	< 10	16	< 10	< 10	< 10	10	35	4
113		520	150	250	50	54	220	10	22	1, 2, 3
114		47	38	66	100	< 10	< 10	< 10	< 10	1, 2
115		< 10	10	72	700	< 10	10	< 10	< 10	2
116		> 1,000	> 1,000	680	> 1,000	700	> 1,000	80	70	1, 2, 3, 4
117		> 1,000	92	460	170	< 10	29	23	27	1, 2
118		700	520	< 10	48	< 10	< 10	< 10	< 10	1, 2
119		980	240	680	> 1,000	35	62	47	110	1, 2, 3, 4
120		130	< 10	86	100	< 10	< 10	< 10	35	1, 2, 4
121		> 1,000	820	72	10	< 10	< 10	< 10	< 10	1, 2
122		33	10	16	33	88	32	< 10	17	1, 2, 3
123		150	110	< 10	12	< 10	< 10	< 10	< 10	1
124		80	37	210	88	200	270	< 10	19	1, 2, 3
125		72	18	440	74	14	28	< 10	13.5	1, 2
126		> 1,000	84	490	220	< 10	40	< 10	10	1, 2, 3
127		> 1,000	14	> 1,000	> 1,000	84	780	30	52	1, 2, 3, 4
128	36–40	< 10	< 10	92	220	< 10	< 10	< 10	< 10	2
129		820	420	49	11.5	< 10	12.5	< 10	< 10	1, 2
130		40	< 10	47	< 10	19	66	< 10	< 10	1, 2, 3
131		140	34	340	160	< 10	11	< 10	21	1, 2
132		140	74	22	21	< 10	11	< 10	< 10	1
133		1,000	390	300	100	50	97	< 10	50	1, 2, 3, 4
134		340	90	< 10	19	< 10	< 10	< 10	< 10	1

(continued)

Supplemental Table 1

Continued

Serial number	Age group	PRNT$_{50}$ titer								Results
		DENV-1		DENV-2		DENV-3		DENV-4		
		EHI0650Y08	EDEN203/05	EHI1170Y08	EHI0866Y07	EDEN219/05	EHI0040Y09	TCR310A129	EHI462Y04	
135		160	41	270	86	11.5	24	< 10	< 10	1, 2
136		13	< 10	160	54	< 10	< 10	< 10	< 10	2
137		580	430	70	50	< 10	11	< 10	< 10	1, 2
138		< 10	< 10	64	190	< 10	< 10	< 10	< 10	2
139		190	110	82	420	120	160	22	34	1, 2, 3, 4
140		34	< 10	33	21	< 10	< 10	19	28	1, 2
141		230	36	135	120	115	440	27	140	1, 2, 3, 4
142		34	< 10	22	< 10	< 10	60	< 10	< 10	1, 3
143		220	40	160	44	< 10	20	< 10	16	1, 2
144		58	38	250	900	11.5	29	< 10	11	1, 2
145		300	< 10	340	56	120	740	66	56	1, 2, 3, 4
146		23	< 10	50	190	< 10	< 10	< 10	36	2, 4
147		19	< 10	120	320	< 10	19	< 10	15	2
148		68	50	> 1,000	> 1,000	< 10	10	10	36	1, 2, 4
149		42	< 10	290	860	< 10	< 10	< 10	< 10	1, 2
150		< 10	< 10	29	64	< 10	< 10	< 10	58	2, 4
151		23	12	62	< 10	< 10	< 10	< 10	< 10	2
152		35	44	185	560	24	50	< 10	< 10	1, 2, 3
153		125	< 10	1,000	> 1,000	< 10	< 10	< 10	< 10	1, 2
154		16	< 10	68	600	< 10	< 10	< 10	< 10	2
155		430	360	600	780	62	130	13	24	1, 2, 3
156		680	105	74	32	< 10	< 10	< 10	10	1, 2
157		> 1,000	540	420	840	36	74	< 10	44	1, 2, 3, 4
158	56–60	54	26	110	470	< 10	13	< 10	< 10	1, 2
159		19	< 10	120	39	< 10	< 10	< 10	< 10	2
160		< 10	< 10	310	110	< 10	< 10	36	35	2, 4
161		35	42	49	< 10	< 10	< 10	< 10	< 10	1, 2
162		38	< 10	115	42	< 10	< 10	< 10	18	1, 2
163		200	10	150	74	70	200	15	27	1, 2, 3
164		27	< 10	190	37	< 10	11	< 10	29	2
165		78	< 10	200	120	30	140	15	26	1, 2, 3
166		620	32	430	220	< 10	120	< 10	28	1, 2, 3
167		56	11	60	35	80	220	24	15	1, 2, 3
168		170	110	100	440	30	96	50	140	1, 2, 3, 4
169		200	105	150	220	110	100	10	40	1, 2, 3, 4
170		680	270	600	110	47	41	17	60	1, 2, 3, 4
171		520	105	450	> 1,000	15	39	10	38	1, 2, 3, 4
172		160	28	100	< 10	64	88	30	50	1, 2, 3, 4
173		115	21	220	56	100	19.5	< 10	11	1, 2, 3
174		270	140	120	64	10	34	< 10	20	1, 2, 3
175		340	64	82	76	< 10	15	11	40	1, 2, 4
176		940	23	880	76	130	290	31	13	1, 2, 3, 4
177		170	< 10	140	16	< 10	30	< 10	< 10	1, 2, 3
178		150	74	36	26	< 10	25	< 10	< 10	1, 2
179		72	< 10	110	72	< 10	14	< 10	14	1, 2
180		> 1,000	> 1,000	> 1,000	> 1,000	36	76	< 10	31	1, 2, 3, 4
181		330	35	450	90	64	150	< 10	10	1, 2, 3
182		540	49	360	440	62	450	76	84	1, 2, 3, 4
183		360	72	330	340	160	300	12.5	21	1, 2, 3
184		220	10	120	36	86	100	< 10	17	1, 2, 3
185		> 1,000	> 1,000	280	250	> 1,000	> 1,000	12	23	1, 2, 3
186		740	310	370	200	< 10	16	14	50	1, 2, 4
187		96	32	105	32	42	200	< 10	< 10	1, 2, 3

DENV = dengue virus; IgG = immunoglobulin G; PRNT = plaque reduction neutralization test.
Antibody titers were expressed as the reciprocal of the end point dilution.
Samples with PRNT$_{50}$ titers ≥ 30 were considered as having neutralizing antibody against DENV.
